# Thiazolidinediones Promote Axonal Growth through the Activation of the JNK Pathway

**DOI:** 10.1371/journal.pone.0065140

**Published:** 2013-05-31

**Authors:** Rodrigo A. Quintanilla, Juan A. Godoy, Ivan Alfaro, Deny Cabezas, Rommy von Bernhardi, Miguel Bronfman, Nibaldo C. Inestrosa

**Affiliations:** 1 Centro de Envejecimiento y Regeneración (CARE), Departamento de Biología Celular y Molecular, Facultad de Ciencias Biológicas, Pontificia Universidad Católica de Chile, Santiago, Chile; 2 Laboratorio de Neurociencias, Departamento de Neurología, Escuela de Medicina, Pontificia Universidad Católica de Chile, Santiago, Chile; INSERM U894, France

## Abstract

The axon is a neuronal process involved in protein transport, synaptic plasticity, and neural regeneration. It has been suggested that their structure and function are profoundly impaired in neurodegenerative diseases. Previous evidence suggest that Peroxisome Proliferator-Activated Receptors-**γ** (PPAR**γ** promote neuronal differentiation on various neuronal cell types. In addition, we demonstrated that activation of PPAR**γ**by thiazolidinediones (TZDs) drugs that selectively activate PPAR**γ** prevent neurite loss and axonal damage induced by amyloid-β (Aβ). However, the potential role of TZDs in axonal elongation and neuronal polarity has not been explored. We report here that the activation of PPARγ by TZDs promoted axon elongation in primary hippocampal neurons. Treatments with different TZDs significantly increased axonal growth and branching area, but no significant effects were observed in neurite elongation compared to untreated neurons. Treatment with PPARγ antagonist (GW 9662) prevented TZDs-induced axonal growth. Recently, it has been suggested that the c-Jun N-terminal kinase (JNK) plays an important role regulating axonal growth and neuronal polarity. Interestingly, in our studies, treatment with TZDs induced activation of the JNK pathway, and the pharmacological blockage of this pathway prevented axon elongation induced by TZDs. Altogether, these results indicate that activation of JNK induced by PPARγactivators stimulates axonal growth and accelerates neuronal polarity. These novel findings may contribute to the understanding of the effects of PPARγ on neuronal differentiation and validate the use of PPARγ activators as therapeutic agents in neurodegenerative diseases.

## Introduction

Neurons are one of the most highly polarized cell types, their processes being divided morphologically and functionally into two distinct parts, the axon and dendrites [1], [2]. Axon and dendrites are distinguished from each other by their different membrane and protein composition, length, and function [3], [4]. Interestingly, it has been shown that the shortening and loss of axons are common pathological features of neurodegenerative diseases [5], [6]. Growing evidence suggest that axonal impairment may be involved in the neuronal dysfunction reported in neurodegenerative diseases, including Alzheimer's disease (AD), Parkinson, and Huntington's disease (HD) [5].

Peroxisome Proliferator-Activated Receptor-γ (PPARγ) is a member of the family of transcription factor of PPARs. It has been demonstrated to play an important role in the regulation of cell differentiation in several cells, such as adipocytes and macrophages [7], [8]. An important role of PPARγ in the differentiation of rat mesangial, human trophoblast, and clonal neuronal cells has been demonstrated [9], [10]. PPAR**γ** is expressed in the central nervous system [11], [12], and 15-deoxy-PGJ2, a natural PPARγ ligand stimulates differentiation of pheochromocytoma 12 (PC12) and human neuroblastoma cells [13]. Interestingly, significant defects in brain development have been reported in PPARγ −/− and PPARγ +/− mice, indicating the important role of PPARγ in neuronal development [14]. Previously, we reported that PPARγ is present in rat hippocampal neurons and that its activation by thiazolidinediones (TZDs), including rosiglitazone (RGZ), ciglitazone (CGZ), and troglitazone (TGZ), PPARγ activators that have been routinely used for treatment of diabetes type 2 [15], prevented axon degeneration, neurite loss, and mitochondrial impairment induced by Aβ [11], [12]. More importantly, previous studies showed that treatment with PPARγ agonists induced neurite elongation in PC12 cells, and this event was produced by the activation of Mitogen activated kinase-c-Jun N-terminal kinase (MAPK-JNK) pathway [16]. However, the possible role of PPAR**γ** pathway and JNK on axonal elongation is unknown.

JNK is a member of the mitogen-activated protein (MAP) kinase family [17]. Because of its activation during cellular stress, JNK has been studied extensively as a stress-activated protein kinase. However, it is clear that JNK plays other important roles in neuronal development [17], [18]. JNK signaling has been implicated in the development of cerebellar granule neurons [19]. Mice null for the Jnk1 gene exhibit abnormalities in axonal tracts [18]. Furthermore, mice null for both Jnk1 and Jnk2 exhibit severe neurological defects and die during embryogenesis [20]. Recent studies support a role of JNK in the regulation of neurite outgrowth during development [21], [22]. JNK has also been implicated in regulating transcriptional events that regulate neurite outgrowth in PC12 cells [23] and axon regeneration in dorsal root ganglion neurons [24], [25]. More importantly, Oliva et al., showed that inhibition of JNK activity by pharmacological or molecular approaches block axonogenesis but does not inhibit neurite formation or prevent dendritic differentiation [21].

Here, we describe the effect of several PPARγ agonists in neurite and axonal elongation of hippocampal neurons. We found that PPARγ activation promotes axon elongation by a mechanism that involved JNK activation. Treatment with TZDs significantly increased axonal growth and the use of PPARγ antagonists like GW 9662, abolished axonal elongation induced by TZDs. Neurite outgrowth was not significantly increased by treatment with TZDs, indicating that PPARγ-induced effects are particularly strong on axonal growth. Pharmacological inhibitors of JNK pathway prevented TZDs-induced axonal elongation, and more importantly, activation of PPARγsignificantly increased JNK activation on hippocampal neurons.

Altogether, these results suggest a novel role of PPARγ participating in axogenesis and neuronal polarity mediating activation of JNK. These observations extend previous studies that showed a protective role of PPARγ in neurodegenerative diseases and validate a potential use of PPARγ activators against the neuronal damage observed in neurodegenerative diseases.

## Experimental Procedures

### 2.1. Materials

Chemicals, culture media and serum were obtained from Sigma (St. Louis, MO), Roche (Alameda, CA), Merck (Darmstadt, Germany), Gibco BRL (Paisley, UK) and Calsein AM from Molecular Probes (Leiden, The Netherlands). Troglitazone (TGZ), rosiglitazone (RGZ), ciglitazone (CGZ), and GW-9662 (GW) were obtained from Cayman Chemical (Ann Arbor, MI). The antibody anti-tau-1 was kindly donated by Dr. Alejandra Alvarez (Faculty of Biological Sciences, Pontificia Universidad Católica de Chile), antibodies: anti-PPAR**γ**, anti-total JNK, anti-p-JNK, anti-neurofilament, and anti-p-Extracellular signal response kinase (p-ERK) antibodies were from Santa Cruz Biotechnology (San Diego, CA).

### 2.2. Ethics statement

Sprague-Dawley rats used in these experiments were housed at the Faculty of Biological Sciences of the Pontificia Universidad Católica de Chile and handled according to guidelines outlined and approved by the Institutional Animal Care and Use Committee at the Faculty of Biological Sciences of the Pontificia Universidad Católica de Chile.

### 2.3 Primary rat hippocampal culture

Hippocampi from Sprague-Dawley rats at embryonic day 18 were dissected, and primary hippocampal cultures were prepared as previously described [26], [27]. Pregnant dams (18 days) were anesthetized with CO_2_ before obtaining the 18-day rat embryos used for the hippocampal cell cultures. All procedures were performed in agreement with the animal handling and bioethical requirements established by Institutional Animal Care and Wellbeing Committee at the Faculty of Biological Sciences of the Pontificia Universidad Católica de Chile. Hippocampal neurons were seeded in poly-L-lysine-coated wells. Then, cultured hippocampal neurons were treated with PPARγ agonists: TGZ (10 µM), RGZ (10 µM), and CGZ (10 µM) for 24, 48, and 72 h. During treatment, hippocampal neurons were observed and images were taken using video microscopy.

### 2.4 Immunofluorescence studies

Hippocampal neurons plated on poly-L-lysine-coated glass coverslips (25,000 cells/cover) and after treatment with the indicated conditions, were immunostained using: anti-PPARγ (1∶500), anti-Tau 1 (1∶1000) and anti-p-JNK (1∶1000) antibodies. Neurons were analyzed using a Zeiss Pascal Confocal microscope (Carl Zeiss, Germany), and morphometric analyses were carried out using Image-Pro plus software (Mediacybernetics, Bethesda, MD).

### 2.5 Cell fractionation and Western blot analysis

After indicated treatments, hippocampal neurons were homogenized, and centrifuged at 100,000× g at 4°C for 1 h. Supernatants were collected and analyzed by 10% SDS-PAGE. Protein bands were transferred to nitrocellulose membranes, and detected with appropriate primary antibodies [28], [29].

### 2.6 Morphometric analysis

Hippocampal neurons plated on poly-L-lysine-coated covers (25,000 cells/cover) treated with PPARγ agonists were observed from time 0 to 72 h, and neuronal development was followed using a Zeiss Axiovision fluorescence microscope equipped with a culture chamber (37°C and 5% CO_2_) and video recording system [27]. The following neurite morphology parameters were evaluated: axonal length, length of minor processes (neurites) and neuronal polarity. For the analysis, an axon-like neurite was defined as a process at least twice as long as the other neurites of the same cell, with a minimum length of 50 µm [30]. A total of 200 cells from 3 independent hippocampal cultures were analyzed for each experimental condition and time point. Additionally, using the same protocol described above, we immunolabeled hippocampal neurons exposed to the different experimental conditions with monoclonal anti-tau-1 antibody (1∶500), or loaded neurons with Calsein AM dye (Molecular Probes), in order to evaluate morphometric parameters. Neuronal complexity analysis (Scholl analysis) was made according to Codocedo et al. [30]. Scholl analysis is a quantitative measure of the size and shape of the dendritic tree [31]. In our studies, it represents a measure of how axon length is changing in relation of neuronal soma [30]. The total length of axons and neurites were quantified using Image-Pro plus software as previously described [11], [26]. Differences among groups were evaluated by the analysis of variance and Student-Newman-Keuls test.

### 2.7 Wnt 5A conditioned Medium

Wnt 5A conditioned medium was generated according to Farias et al [32]. Briefly, human embryonic kidney 293 (HEK- 293) cells were transiently transfected by calcium phosphate precipitation with an empty vector pcDNA (control) or a pcDNA containing sequences encoding for Wnt 5A constructs [32]. The presence of Wnt-5A ligands in the conditioned medium was verified by Western blot analysis using an antibody against the hemagglutinin (HA) epitope [32].

### 2.8 Statistical analysis

Results were expressed as the mean ± standard error (S.E.M.). Differences among groups were evaluated by analysis of variance and Student-Newman-Keuls test. Students t test was used for analyzing data for Western blot and image analysis. P<0.05 was regarded as statistically significant.

## Results

### 3.1. PPARγ activation promotes axonal elongation on hippocampal neurons

**Figure 1:**
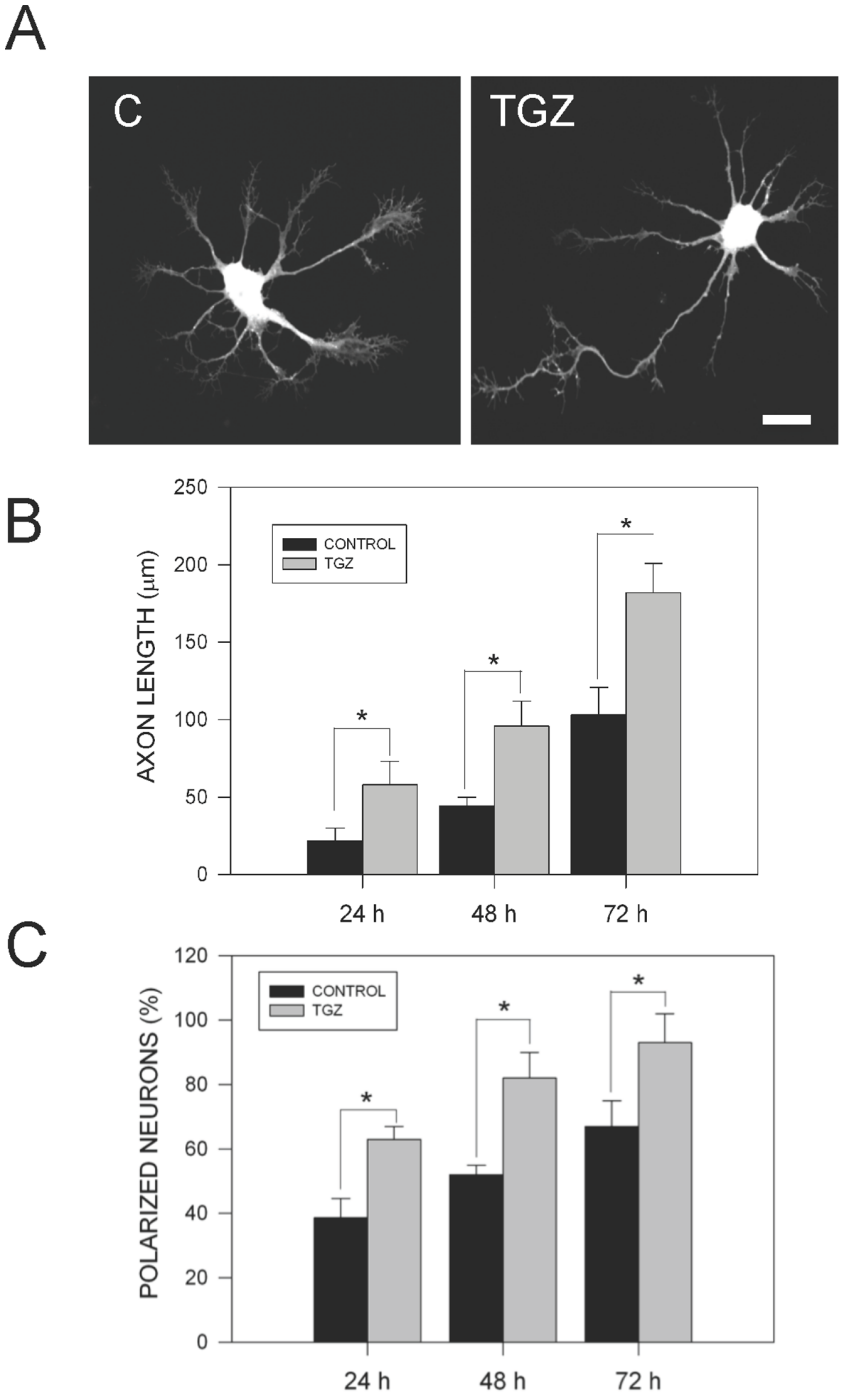
PPARγ agonists (TZDs) promote axonal growth in hippocampal neurons. (**A**) Representative confocal images of primary hippocampal neurons loaded with calsein AM for 30 min, that were untreated (C) or treated with 10 µM troglitazone (TGZ) for 24 h. TGZ significantly increased axonal length compared with untreated neurons. (**B**) Quantification of average axonal length of hippocampal neurons treated with TGZ for 24, 48, and 72 h. Data correspond to the mean ± S.E.M. (bars) from 4 independent experiments, *p<0.05. (**C**) Quantification of polarized neuronal population in neurons untreated (C) and treated with TGZ for the indicated times. Measurement indicates the% of neurons that present a defined axon. Data are the mean ± S.E.M. (bars) from 4 independent experiments, *p<0.01.

**Figure 2:**
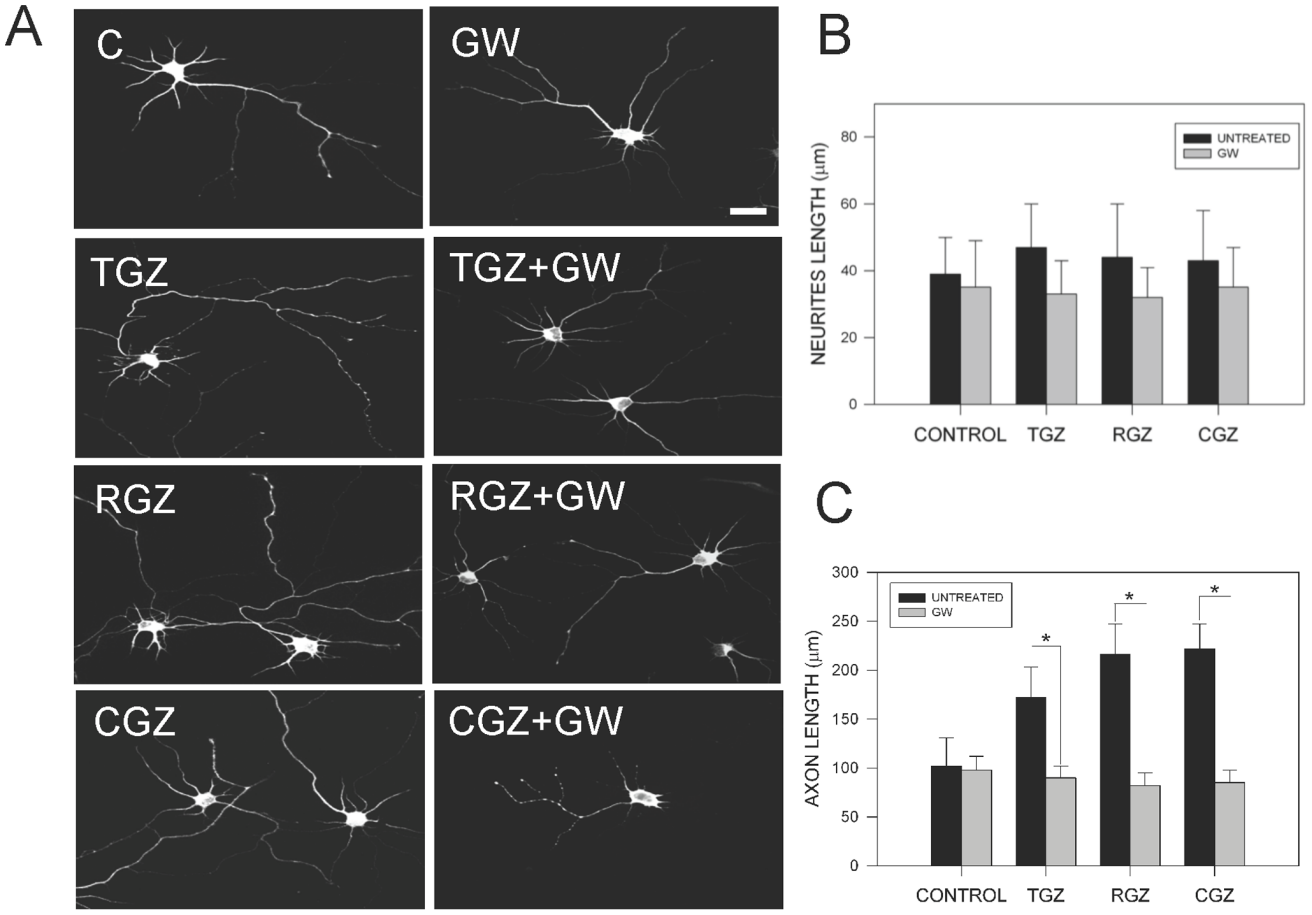
Blockage of PPARγ activation prevented the increase of axonal growth induced by TZDs. (**A**) Confocal images from hippocampal neurons that were treated with PPARγactivators: TGZ (10 µM), RGZ (10 µM), and CGZ (10 µM) for 72 h, in the presence or absence of 5 µM GW 4622 (GW). GW is a specific inhibitor of PPARγ activity [11]. After treatment hippocampal neurons were fixed and immunolabeled with anti-tau-1 antibody and then were photographed in a confocal microscope. (**B**) Quantification of total neurite length on hippocampal neurons exposed to TZDs, in the presence or absence of GW for 72 h. Treatment with PPARγ activators plus GW did not significantly affect total neurite length of hippocampal neurons. Data are the mean ± S.E.M. of 4 independent experiments, *p<0.05. (**C**) Quantification of average axonal length in hippocampal neurons treated with TZDs in the presence or absence of GW for 72 h. Data are the mean ± S.E.M. of 4 independent experiments, *p<0.05.

PPARγactivation with TGZ prevents neuronal cell death and calcium stress induced by Aβ peptide [11]. In that study, PPARγ activation by agonists induced an increase of axonal caliber and neurite length on hippocampal neurons [11]. Previous evidence suggests that PPARγ activation promotes neurite extension in PC12 cells exposed to soluble Nerve Growth Factor (NGF) [16]. Treatment with the PPARγ agonist TGZ (10 µM) for 24 h accelerated axonal development on hippocampal neurons (Fig. 1). Similar results were obtained with other PPARγ activators including RGZ (10 µM) and CGZ (10 µM) (Fig. 2A, C). Neuronal development was evaluated measuring axonal growth (Fig. 1B), neuronal polarity (Fig. 1C), and neurite outgrowth (Fig. 2B). Treatment with TGZ induced a two-fold increase in the axonal length compared with untreated neurons (Fig. 1A, B). Additionally, TGZ induced a substantial increase in the percentage of hippocampal neurons showing neuronal polarization (Fig. 1C). We also observed that in hippocampal cultures exposed to TGZ for 72 h, around 98% of the neurons showed a polarized phenotype, which means that they developed a distinguishable axonal process with minor secondary processes (Fig. 1C) [30]. These results suggest that activation of PPARγby TZDs drugs promotes axonal growth and neuronal polarity in rat hippocampal neurons.

### 3.2. Blockage of PPARγ activation prevented the increase in axonal growth in hippocampal neurons treated with TZDs

To corroborate the effects observed with TGZ, we tested other PPARγ activators belonging to the TZDs family, like RGZ and CGZ, and the specific PPARγ antagonist GW 4662 (GW) [15]. TZDs drugs have been used for the treatment of diabetes mellitus type 2 [15], and their use have recently been associated with a significant recovery of memory impairment in Alzheimer's disease patients [33]. GW is an antagonist of the PPARγ receptor. In ours hands, it was capable of preventing neuronal cell death protection induced by TGZ in Aβ-treated neurons [11]. Figure 2 shows the effect of PPARγ agonists in neurite and axonal outgrowth in presence and absence of 5 µM GW. Measurement of total neurite length in hippocampal cultures treated with TZDs plus GW did not show significant differences compared with untreated neurons (Fig. 2B). Further studies in neurons treated with TZDs plus GW showed a significant reduction in axonal length (Fig. 2C). These indications suggest that TZDs-mediated effect were PPARγ-dependent and were mainly observed in the axon. In addition, RGZ and CGZ increased the percentage of polarized neurons, similar to the effect observed after TGZ treatment showed in Figure 1. This effect was also abolished by incubation with GW (data not shown).

### 3.3. PPARγ agonists induced PPARγ expression and its axonal localization in hippocampal neurons

**Figure 3:**
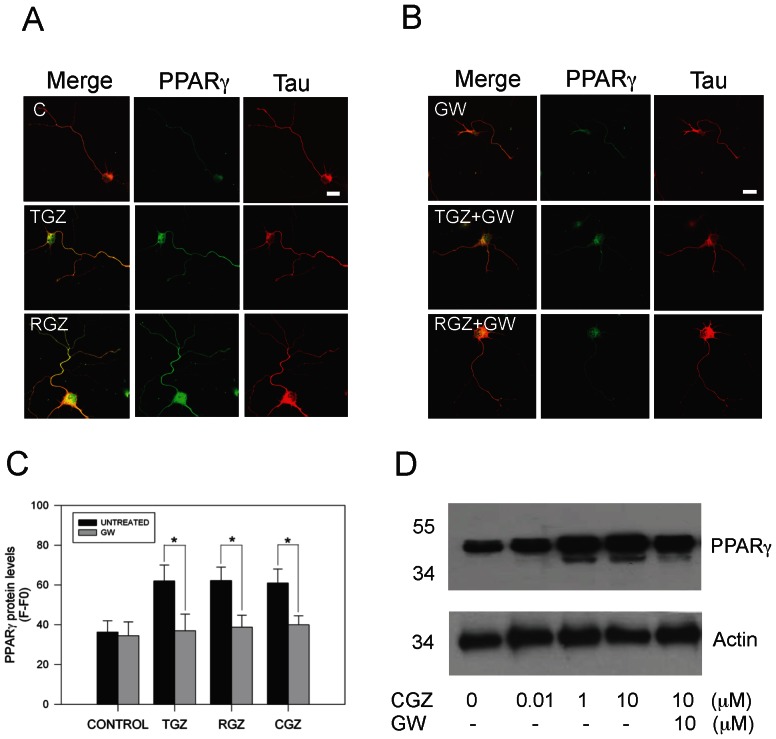
TZDs treatment increased PPARγ level and its axonal localization in hippocampal neurons. (A) Hippocampal neurons were treated with TGZ (10 µM) and RGZ (10 µM) for 72 h. After treatment, neurons were fixed and double immunolabeled with anti-PPARγ (green) and anti-tau-1 (red) antibodies, respectively. Confocal images show that TZDs treatment increased PPARγ protein levels. (**B**) Representative images from neurons treated with TZDs, in the presence or absence of GW, and double labeling with anti-PPARγ and anti-tau-1 antibodies. GW prevented the increase in the PPARγ levels induced by TGZ and RGZ treatments. (**C**) Estimation of PPARγ level by evaluation of fluorescence intensity of PPARγ signal in representative images obtained from hippocampal neurons exposed to TZDs, in the presence or absence of GW for 72 h. Data are the mean ± S.E.M. from 4 independent experiments, *p<0.05. (**D**) Hippocampal neurons in culture treated with increasing CGZ concentrations and 10 µM GW for 72 h, were prepared for detection of PPARγprotein levels by western blot. Representative blot showed that CGZ increased PPARγ and treatment with GW prevented the increase of PPARγ levels induced by CGZ. Actin protein levels are showed as a control. Data are representative of 3 independent experiments.

We evaluated by immunofluorescence protein expression and localization of PPARγreceptor in hippocampal neurons in response to TZDs. Figure 3 shows representative immunofluorescence images and analysis of the levels and distribution of PPARγ in neurons exposed to 10 µM TZDs for 72 h. TZDs induced a robust increase in PPARγ levels, in comparison with untreated neurons (Fig. 3A). Additionally, we observed a significant axonal localization of PPARγ in neurons treated with PPARγ agonists (Fig. 3A). Immunofluorescence studies evidenced a robust and close localization between anti-tau-1 and anti-PPARγ antibody in TZDs-treated neurons. PPARγ staining of untreated neurons predominated in the nucleus with not apparent co-localization between tau-1 and PPARγ in axons (Fig. 3). Interestingly, in hippocampal cultures co-treated with TZDs and 10 µM GW, PPARγ levels were significantly decreased, indicating that the effect of TZDs were mediated by specific activation of PPARγ (Fig. 3B). Quantitated data from representative images of neurons treated with TDZs and immunolabeled for tau-1 and PPARγindicated that PPARγ activation by TZDs significantly increased protein PPARγ levels in hippocampal neurons (Fig. 3C). The immunofluorescence data presented above was corroborated by western blot studies made in hippocampal neurons treated with increasing concentrations of CGZ, and in the presence of GW (Fig. 3D). Treatment with CGZ increased PPARγ protein levels, effect that was prevented by GW (Fig. 3D). These results suggest that PPARγ activation by TZDs increased PPARγ protein levels, and also promoted localization of PPARγin the axon of hippocampal neurons. This effect could facilitate the accelerated axonal growth observed in the TZDs-treated neurons.

### 3.4. Role of JNK in the increase of axonal growth induced by PPARγactivation

**Figure 4:**
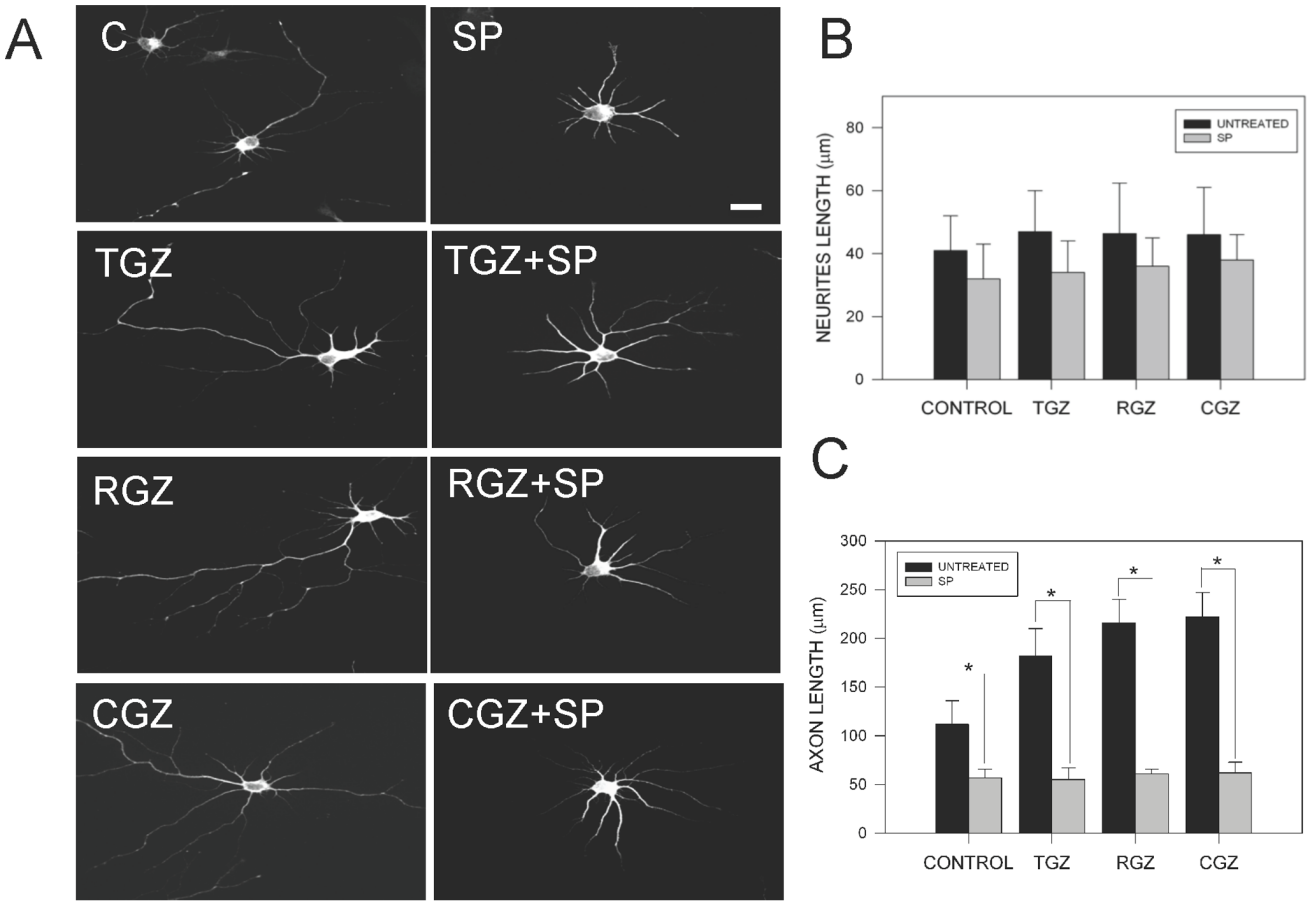
Possible role of JNK on axonal elongation induced by PPARγ activators. (**A**) Representative confocal images of hippocampal neurons treated with various TZDs (10 µM) in the presence or absence of the JNK inhibitor SP600125 (SP, 100 nM) for 72 h. After treatment, neurons were fixed and immunolabeled with anti-tau-1 antibody. SP prevented axonal elongation induced by TZDs without affecting the average neurite length. (**B**) Quantification of total neurite length in hippocampal neurons exposed to TZDs, in the presence or absence of SP for 72 h. Treatment with PPARγ activators plus SP did not significantly affected total neurite length on hippocampal neurons. Data are the mean ± S.E.M. of 4 independent experiments, *p<0.05. (**C**) Quantification of axonal length on hippocampal neurons treated with TZDs in the presence or absence of SP for 72 h. Data are the mean ± S.E.M. of 4 independent experiments, *p<0.05. Axonal length was evaluated using Image Pro software. Data are the mean ± S.E.M. of 4 independent experiments, *p<0.05 and **p<0.01.

**Figure 5:**
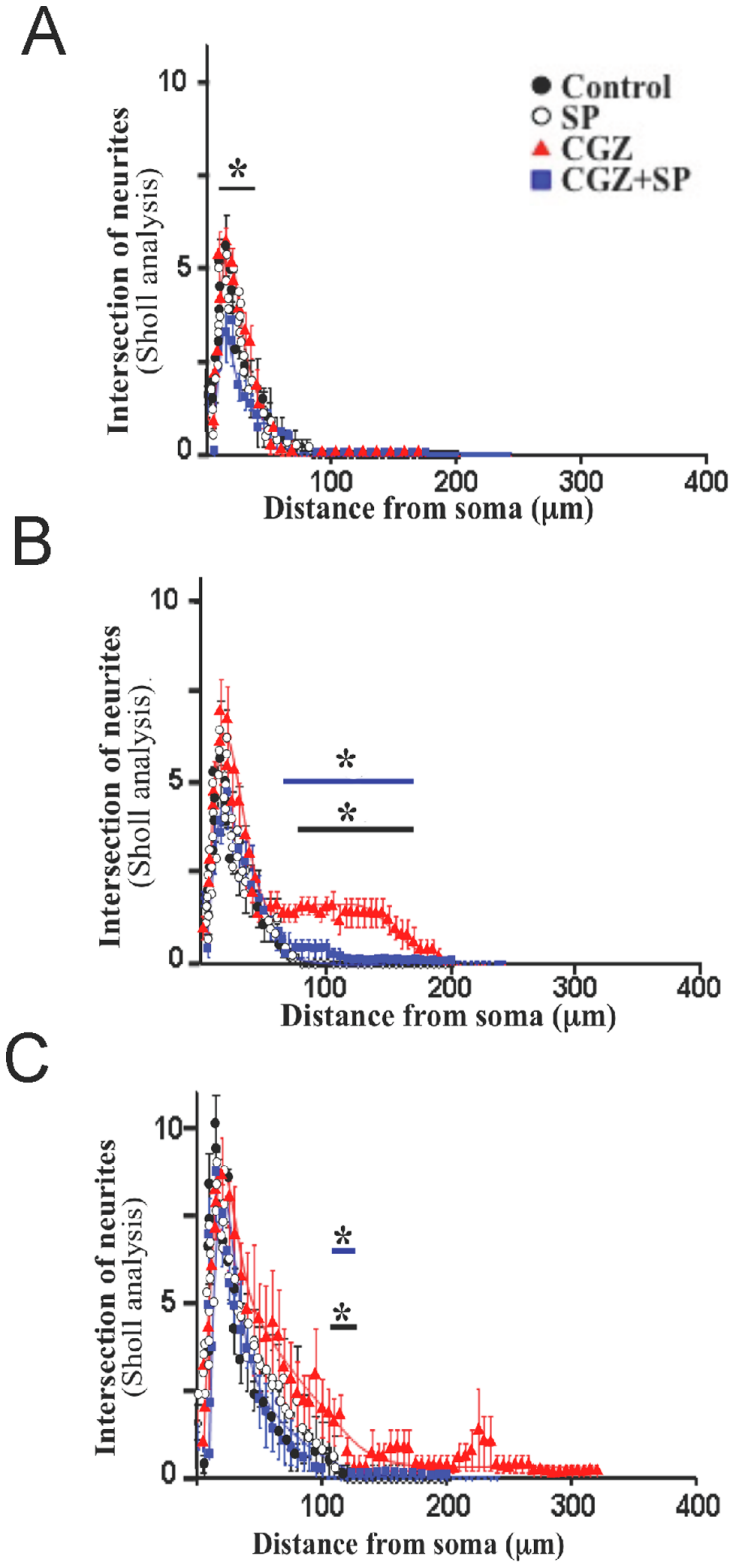
Inhibition of JNK reduced axonal elongation on hippocampal neurons treated with CGZ. Cultured hippocampal neurons were treated with CGZ (10 µM) and CGZ + SP (CGZ, 10 µM + SP, 100 nM) for 24 (**A**), 48 (**B**), and 72 (**C**) h. Representative confocal images labeled with anti-tau 1 antibody were evaluated by the Sholl analysis. Black brackets indicate significant differences between treatment with CGZ and untreated neurons (C), blue brackets indicate significant differences between treatment with CGZ and CGZ plus SP. Data are the mean ± S.E.M. of 4 independent experiments. *p<0.05.

Previous evidence suggests that neurite elongation induced by PPAR**γ** agonists in PC12 cells is produced by activation of MAPK, p38, and JNK kinase [16]. Additionally, studies in knock out mice for JNK showed a delay in neuronal development with evident signs of neurodegeneration [34]. To study the possible role of JNK in TZDs-induced axonal elongation, we studied hippocampal neurons treated with PPARγ agonists (10 µM) in the presence of the specific JNK inhibitor SP 600125 (SP; 100 nM) [17], [32]. Figure 4A shows representative confocal images of neurons exposed to the indicated conditions for 72 h. Inhibition of JNK prevented axonal elongation induced by TZDs (Fig. 4A). The effect was significant only for average axonal length (Fig. 4C). In contrast, quantification of independent experiments did not show statistical differences for neurite total length in neurons treated with PPARγ agonists in presence of SP (Fig. 4B). Additional quantification analysis indicated that TZDs-induced axonal growth was dependent on JNK activation (Figs. 5 and Fig S2). A time course of hippocampal neurons exposed to 10 µM CGZ in the presence or absence of 100 nM SP and labeled with anti-tau 1 antibody to specifically detect the axon, indicated that the increased axonal growth was totally prevented by the JNK inhibitor SP (Fig. S1). Additional analysis of neuronal complexity (Scholl analysis) supports the role of JNK in axonal elongation induced by TZDs (Fig. 5) [30], [31]. Scholl analysis indicated that TZDs treatments clearly induced axon elongation and pre-treatment with SP totally prevented this effect (Fig. 5A, B, C). These results suggest that PPARγ activation promotes axonal elongation by the activation of JNK in hippocampal neurons.

### 3.5. PPARγ agonists induce JNK activation in primary hippocampal neurons

**Figure 6:**
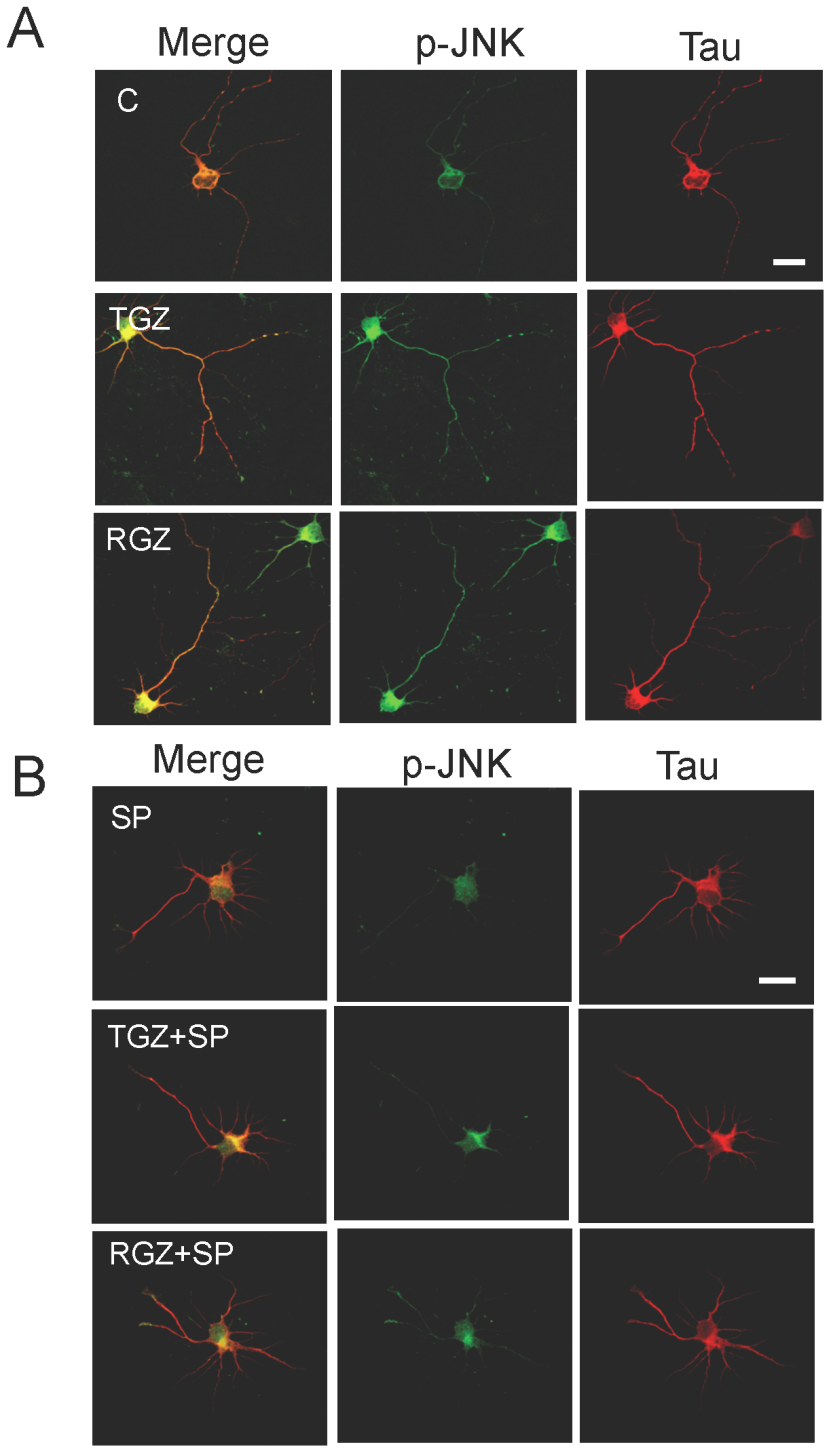
PPARγ agonists induce JNK activation on primary hippocampal neurons. (**A**) Hippocampal neurons were treated with TGZ (10 µM) and RGZ (10 µM), in the presence or absence of SP (100 nM) for 72 h. After treatment neurons were fixed and double immunolabeled with anti-p-JNK (green) and anti-tau-1 (red) antibodies, respectively. Confocal images show that TZDs treatment increases p-JNK levels. (**B**) Representative images from neurons treated with TZDs, in the presence or absence of SP, double labeled with anti-p-JNK and anti-tau-1 antibodies. Inhibition of JNK using SP prevented the increase of p-JNK level induced by TGZ and RGZ, respectively. Representative images were taken from 4 independent experiments.

**Figure 7:**
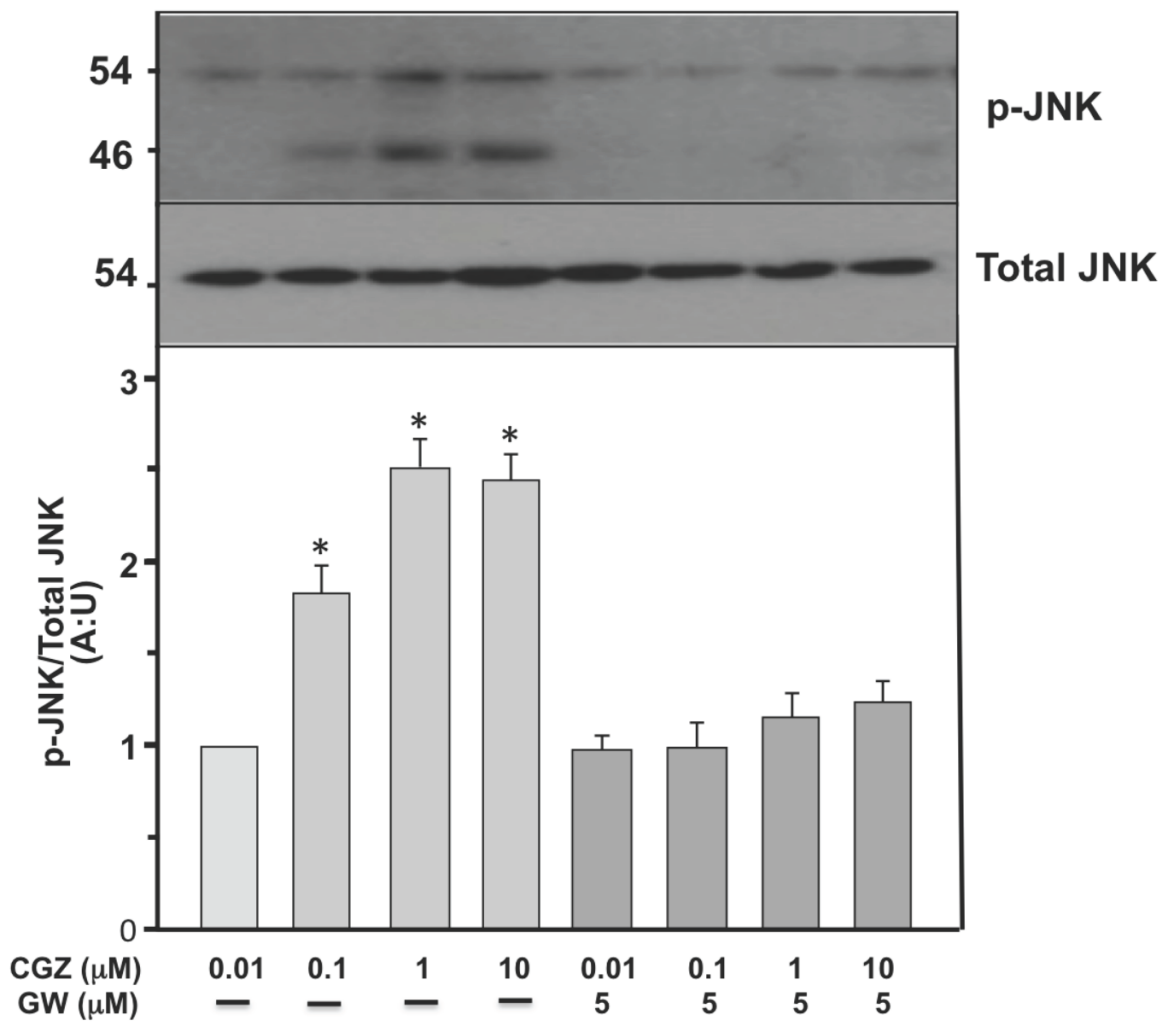
CGZ increases p-JNK levels on primary hippocampal neurons. Cultured hippocampal neurons (15 DIV) were treated with CGZ and GW for 72 h, and cells extracts were prepared for p-JNK and total JNK protein levels detection by western blot. Quantification of 4 independent experiments showed that CGZ increased p-JNK levels with not apparent effect on total JNK. Inhibition of PPARγ with GW prevented the increase of p-JNK induced by CGZ. Data are the mean ± S.E.M. of 4 independent experiments. *p<0.05 show significant differences between untreated compared with CGZ or CGZ plus GW treated neurons.

Figure 6 shows representative confocal images from neurons double labeled with anti tau-1 and anti-phosphorylated JNK (p-JNK) antibodies after being treated with TGZ, RGZ and SP for 72 h. Anti-p-JNK shows the activation of the JNK pathway [29]. There was a strong increase in p-JNK levels in TZDs-treated neurons (Fig. 6A). p-JNK was mainly localized in the axon, suggesting that activation of JNK may participate in axonal elongation induced by TZDs (Fig. 6A). Additionally, immunofluorescence analysis of TZDs-treated neurons showed a conspicuous co-localization of p-JNK and anti-tau 1 labeling (Fig. 6A). As was expected, SP reduced p-JNK levels, and reorganized p-JNK localization towards a cytoplasmic pattern (Fig. 6B). In addition, dose response studies showed that CGZ induced a significant increase in p-JNK expression evaluated by western blot (Fig 7). Interestingly, increased levels of p-JNK were not observed when hippocampal cultures were cultured in the presence of 5 µM GW, suggesting a specific role for PPARγ on the control of JNK activation.

### 3.6 Axonal elongation induced by TZDs is not mediated by external signal response kinase (ERK) activation

**Figure 8:**
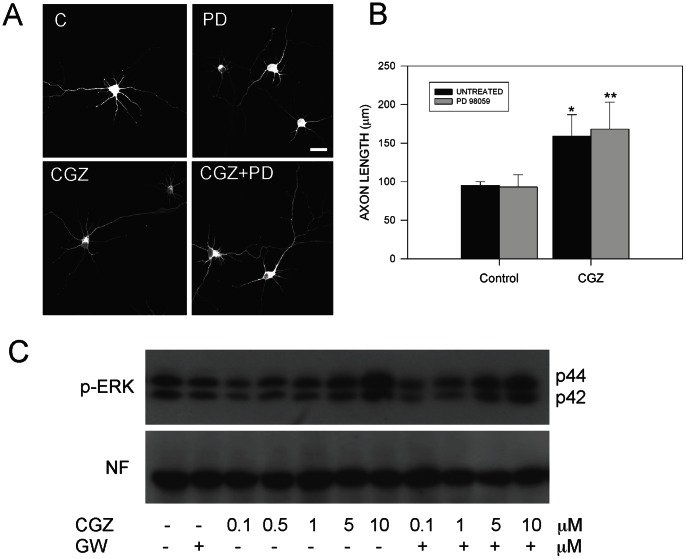
ERK activation is not involved in the axonal elongation induced by TZDs. (**A**) Confocal images from hippocampal neurons that were treated with CGZ (10 µM) for 72 h, in the presence or absence of 5 µM PD 98059 (GW). PD is a specific inhibitor of ERK activation. After treatment, hippocampal neurons were fixed and immunolabeled with anti-tau-1 antibody and photographed in a confocal microscope. (**B**) Treatment with CGZ plus PD did not significantly affect average axonal length. Data are the mean ± S.E.M. of 3 independent experiments. *p<0.05 compared with untreated neurons, **p<0.05 compared with neurons treated with PD 98059. Bar = 10 µm. (**C**) Hippocampal neurons were treated with increasing concentrations of CGZ and GW for 72 h. Cell extracts were prepared for p-ERK and neurofilament (NF) protein levels detection by western blot. A representative blot showed that CGZ increased p-ERK levels with not apparent effect on neurofilament levels. Inhibition of ERK with PD partially prevented the increase of p-ERK induced by CGZ. Data are representative from 3 independent experiments.

In this paper, we show that activation of PPARγ receptors by TZDs enhances axon growth through JNK activation. However, it was previously suggested that PPARγ activators induced neurite outgrowth of PC12 cells [16] and differentiation of embryonic midbrain cells [35] by participation of JNK, p38, and ERK [16], [35]. To study the possible role of ERK in the increase of axon growth produced by TZDs, we treated hippocampal neurons with PPARγ activators in the presence and absence of 5 µM PD 98059 (PD), which is a well-know inhibitor of ERK [32]. Figure 8A shows representative confocal images of hippocampal neurons untreated and treated with 10 µM CGZ and CGZ+PD during 72 h, and immunostained against tau-1 (Fig. 8A). These studies revealed that inhibition of ERK has not apparent effect on the axonal elongation induced by CGZ (Fig. 8A, B). In addition, we evaluated the activation levels of ERK in hippocampal neurons treated with increasing concentrations of CGZ in the presence of GW (Fig. 8C). Western blot studies indicated that treatment with 10 µM CGZ significantly increased p-ERK levels compared with untreated neurons (Fig. 8C). However, inhibition of PPARγ activation by GW was not able to prevent p-ERK levels increased by CGZ (Fig. 8C). These studies suggest that ERK is not participating in the increased axonal growth produced by TZDs in hippocampal neurons.

### 3.7 Treatment with ligand Wnt 5A and TGZ increased axon growth through the JNK pathway

**Figure 9:**
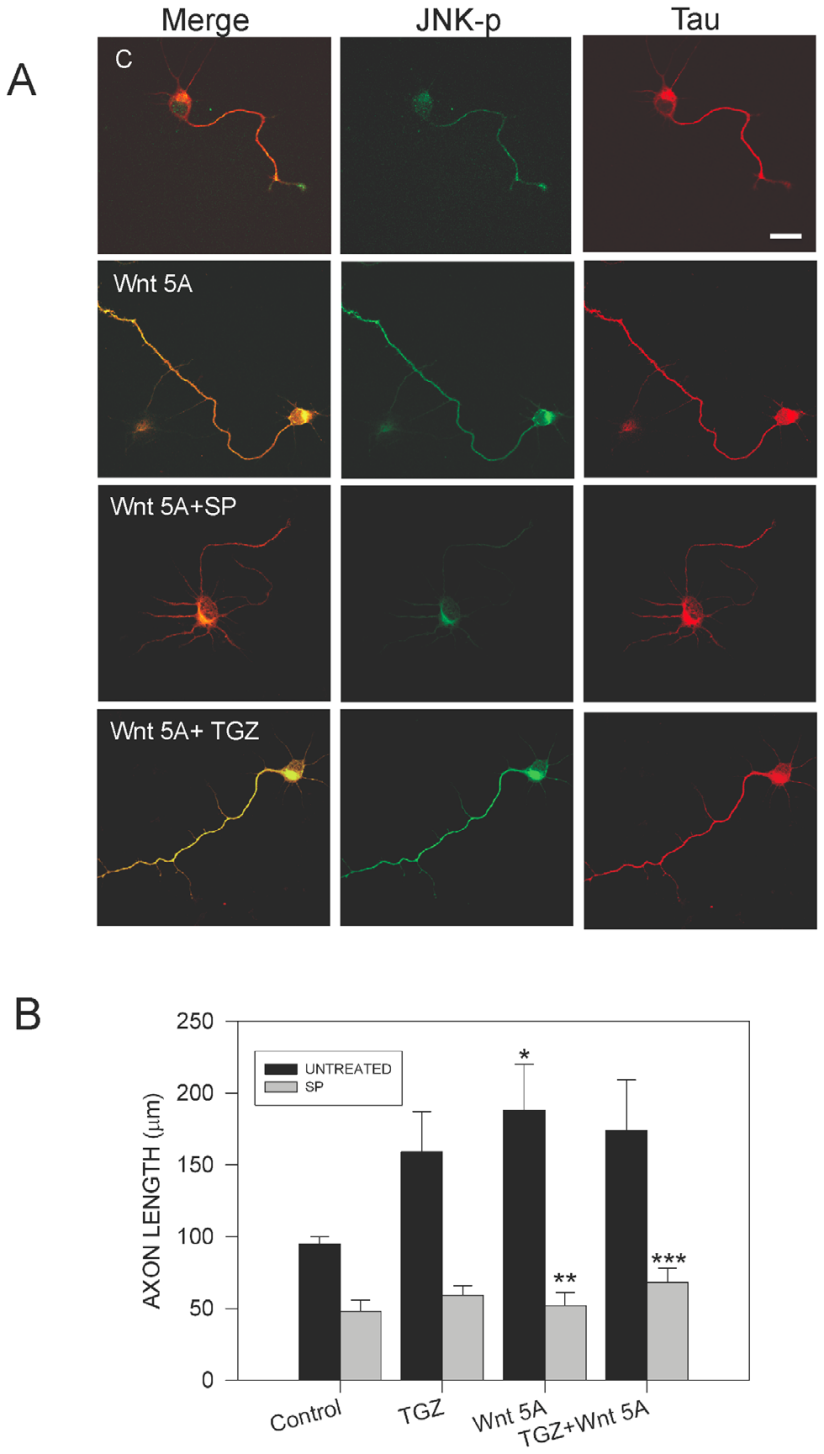
Wnt 5A and TGZ increases axonal elongation through JNK pathway. (**A**) Representative confocal images of hippocampal neurons treated with Wnt 5A conditioned medium and 10 µM TGZ in the presence or absence of the JNK inhibitor SP600125 (SP, 100 nM) for 72 h. After treatment neurons were fixed and immunolabeled with anti-tau-1 and anti-p-JNK antibodies. Treatment with Wnt 5A and TGZ significantly increased axon growth. SP prevented axonal elongation induced by Wnt 5A and TGZ. Inhibition of JNK by SP prevented the increase of p-JNK level induced by Wnt 5A and TGZ, respectively. Representative images were taken from 4 independent experiments. (**B**) Quantification of axonal length on hippocampal neurons treated with Wnt 5A and TGZ in the presence or absence of SP for 72 h. Data are the mean ± S.E.M. of 4 independent experiments. *p<0.05 compared with untreated neurons, ** *p*<0.05 compared with Wnt 5A treated neurons, *** *p*<0.05 compared with Wnt 5A+ TGZ treated neurons.

Wnt proteins are morphogens that play important roles during embryogenesis [36]. Wnt proteins signal through at least two different pathways: canonical and non-canonical [32], [36]. In the canonical pathway, Wnt signals through Dishevelled (Dvl) to increase cytoplasmicβ-catenin levels, and then β-catenin enters the nucleus, where it co-activates transcription of Wnt target genes [36]. Non-canonical Wnt signaling pathways mediate several cellular processes through different molecular intermediates, including Rho-GTPases, intracellular calcium levels and JNK activation [32]. Recently, it has been shown that the ligand Wnt 5A, an activator of non-canonical Wnt pathway, could play a role in the process of axonal growth and guidance [37], [38]. Treatment with Wnt 5A increased axon outgrowth and enhances the vesicle transport to growth cones in cortical neurons [38]. In addition, we previously reported that treatment with Wnt 5A rapidly induced activation of JNK pathway [32]. However, the mechanism for the participation of Wnt 5A in axon elongation is not completely elucidated. Therefore, we treated hippocampal neurons with conditioned medium containing Wnt 5A during 72 h, and then neurons were fixed and double staining with anti-tau1 and anti-p-JNK antibodies, and axon length was analyzed (Fig. 9). Representative confocal images showed that treatment with Wnt 5A significantly increased axonal elongation compared with untreated neurons (Fig. 9A, B). Interestingly, axonal growth increase by Wnt 5A was abolished in the presence of JNK inhibitor SP, suggesting that JNK could be involved in this process (Fig. 9A, B). As we previously observed in this paper, treatment with TZDs induced axonal elongation through JNK pathway (Figs. 4, 5). Therefore, we evaluated axon length in hippocampal neurons treated for 72 h with both Wnt 5A and TGZ. Treatment with Wnt 5A+TGZ induced a significant increase in axonal growth. However, this increase was not significant compared with neurons treated with Wnt 5A or TGZ per separate (Fig. 9B). In addition, p-JNK levels were evaluated in neurons treated with Wnt 5A or Wnt 5A+TGZ, in the presence of SP. Immunofluorescence analysis indicated that Wnt 5A+TGZ treatment for 72 h increased p-JNK levels and this increment was prevented using JNK inhibitor SP. These observations suggest that Wnt 5A and TGZ stimulates axonal growth using a common pathway, in this case, JNK pathway.

Altogether, these observations suggest that JNK kinase plays an important role for axonal elongation induced by PPARγ activators (TZDs) in hippocampal neurons. Both pathways can contribute to neuronal development by promoting the extension of the neuronal processes, and represent a novel therapeutic strategy to promote neuronal protection in neurodegenerative diseases.

## Discussion

Neurite network loss and axonal degeneration has been observed in a wide range of neurodegenerative disorders [5], [6]. These features are common in neurodegenerative diseases, producing anomalous synaptic function, and neuronal cell death [5], [39], [40]. Aβ peptide induces a severe neurite network loss and axonal degeneration in different neuronal cell types [41]. Therefore, it is important to understand how these neurodegenerative changes evolve in order to design new strategies to repair the loss of connections. Here, we showed that PPARγ activation promoted axonal growth in rat hippocampal neurons, effect that was mediated by the activation of JNK kinase induced by activation of PPARγ. Previous studies indicate that PPARγ activation is involved in differentiation of adipocytes and oligodendrocytes [10], [14]. Our findings are in agreement with increased evidence that suggest that PPARγ has a role in neuronal repair [42], [43]. TZDs drugs (e.g., TGZ, RGZ, and CGZ) are PPARγ agonists that increase peripheral insulin sensitivity and stimulate mitochondrial biogenesis and function [15], [44]. Recently, clinical trials showed that pioglitazone improved memory and cognition in a subset of AD patients [33] as well as reduced learning and memory deficits in a mouse model for AD [45]. In addition, other studies describe that PPARγ activation protects from neuronal ischemia, glutamate toxicity, and long terminal potential (LTP) impairment in an AD mice model overexpressing APP protein [46]. Moreover, we showed that PPARγ activation prevents Aβ neurotoxicity effects [11], [12], and RGZ treatment protected from mitochondrial failure induced by mutant huntingtin expression [44]. PPARγ activation and the induction of peroxisomes prevented neuritic network loss and axonal damage induced by Aβ [28]. In fact, the peroxisome proliferation effect induced by Wy (a peroxisome proliferator) is associated with the activation of the PPARαresponse [28]. PGC1-α, a transcriptional factor involved in mitochondrial biogenesis, is involved in this process [47], [48]. Additionally, evidence indicates that PGC1-α could be playing a role in the pathogenesis of Huntington Disease (HD), evidence that support the importance of PPARγ receptor in the neuropathological mechanisms of various neuronal disorders [48], [49].

These events are in agreement with our findings that led us to propose a role for PPARγ activation on the promotion of neuronal development, especially on axonal elongation. TZDs treatment promoted axonal growth and this effect was totally prevented by GW 4622, a specific PPARγ antagonist. In addition, co-treatment with the JNK inhibitor SP600125 prevented axonal elongation induced by TZDs, further supporting the participation of PPARγ pathway. Previous evidence suggests that PPARγis involved in PC12 differentiation induced by nerve growth factor (NGF) through activation of MAPK and JNK [9], [14], [16]. Interestingly, Brodbeck et al. showed that treatment with RGZ significantly increased dendritic spine density in a dose-dependent manner in primary cortical rat neuron cultures [42]. This effect was abolished by GW9662, suggesting that RGZ exerts its effect by activating the PPARγ pathway [42]. Our observations are in agreement with these studies and confirm the potential role of PPARγ promoting neuronal development and synaptic regeneration, by increasing axonal length and dendritic spine density in hippocampal neurons.

Our results suggest that PPARγ promoted axonal elongation by the activation of JNK kinase. There are interesting observations that associate the JNK pathway with neuronal polarity [4], [21]. JNK activity is maintained at an extremely high level in the embryonic brain compared with other MAP kinase-related enzymes [4]. Previous studies show severe impairments on dendritic structure in the cerebellum and motor cortex of c-Jun N-terminal kinase 1 (JNK1)-deficient mice [34]. JNKs may influence cytoskeletal reorganization via the phosphorylation of proteins regulating microtubule stability, including doublecortin (DCX), stathmin family protein (SCG10), and microtubule- associated proteins, MAP2 and MAP1B [34], [50].

Interestingly, it has been shown that activated JNK is required for axonogenesis but not for the formation of minor processes or development of dendrites in hippocampal neurons [21]. Pharmacological blockage of JNK pathway inhibited axonal elongation resulting in a phenotype that may lack a defined axon [21]. In our studies, inhibition of JNK significantly prevented axonal elongation induced by TZDs and the phenotype showed by hippocampal neurons resembled that described by Oliva et al. [21]. Therefore, activation of JNK pathway appears to mediate induction of axonal growth by PPARγ.

In addition, evidence indicates that activating transcription factor-2 (ATF-2) is involved in axonal elongation induced by JNK [21]. JNK can phosphorylate several targets [51], including ATF-2 (also known as CRE-BP1, CREB2, mXBP). ATF-2 is a member of the ATF/CREB (cAMP response element-binding protein), a family of transcription factors that binds to CRE (cAMP-responsive element) and regulates numerous neuronal genes [51], [52]. Interestingly, significant levels of phosphorylated ATF-2 (phospho-ATF-2) were found in the axon, in parallel with the enrichment of p-JNK [21]. In addition, chronic or acute treatment with SP600125 (the former of which inhibited axon formation) decreased phospho-ATF-2, respectively, but did not significantly affect total ATF-2 levels [21].

It has been shown that ATF-2 is required for maximal and accurate PPARγ transcription [51], [53]. ATF-2 directly binds to the PPARγ promoter and activates their transcription to regulate adipocyte differentiation [51]. Therefore, activation of ATF-2 through JNK pathway could be involved in the axonal elongation increase induced by PPARγ agonists in hippocampal neurons. Further studies are required to evaluate ATF-2 involvement in TZDs-induced axonal elongation in hippocampal neurons.

Finally, our work presents evidence that support the role of PPARγ activation through JNK pathway in neuronal development. Combined activation of these two pathways could be beneficial for the promotion of neuroprotective effects in various neurodegenerative disorders.

## Conclusions

Our results suggest that PPARγ stimulation by TZDs induces axonal growth in hippocampal neurons. Treatment with different PPARγ activators significantly increased axonal elongation without effects over other neuronal properties. The use of GW9662, a specific PPARγ antagonist, and SP 600125, an inhibitor of JNK, prevented these changes. Interestingly, other reports show an important role of JNK controlling the neuronal polarity. Our studies showed that JNK activity could be modulated by PPARγ activators, suggesting that the increase in axonal elongation induced by PPARγ agonists is mediated by JNK. Altogether, our results suggest that PPARγ stimulation could contribute to the development and maintenance of a proper neuronal connectivity.

## Supporting Information

Figure S1 **Troglitazone increases axonal elongation in hippocampal neurons.** Hippocampal neurons recently plated were treated with 10 µM troglitazone (TGZ) and axonal development was observed by video microscopy. Neurons were mounted in a culture chamber controlling temperature, CO_2_, and humidity. Images were taken every hour using a cool CCD fluorescence camera (Zeiss, Germany).
(TIFF)

Figure S2 **PPARγ activation increase of axonal elongation is mediated by JNK activation.** Hippocampal neurons treated with CGZ, SP, and CGZ+SP were fixed at the indicated times and immunofluorescence against tau-1 was done. Axonal length was evaluated using Image Pro software. Data are the mean ± S.E.M. of 4 independent experiments, *p<0.05 and **p<0.01.
(TIFF)
