# Supplementary figures and images for: Thiazolidinediones Promote Axonal Growth through the Activation of the JNK Pathway
Source: PLoS One. 2013 May 31;8(5):e65140. doi: 10.1371/journal.pone.0065140 (PMC3669289; doi:10.1371/journal.pone.0065140)

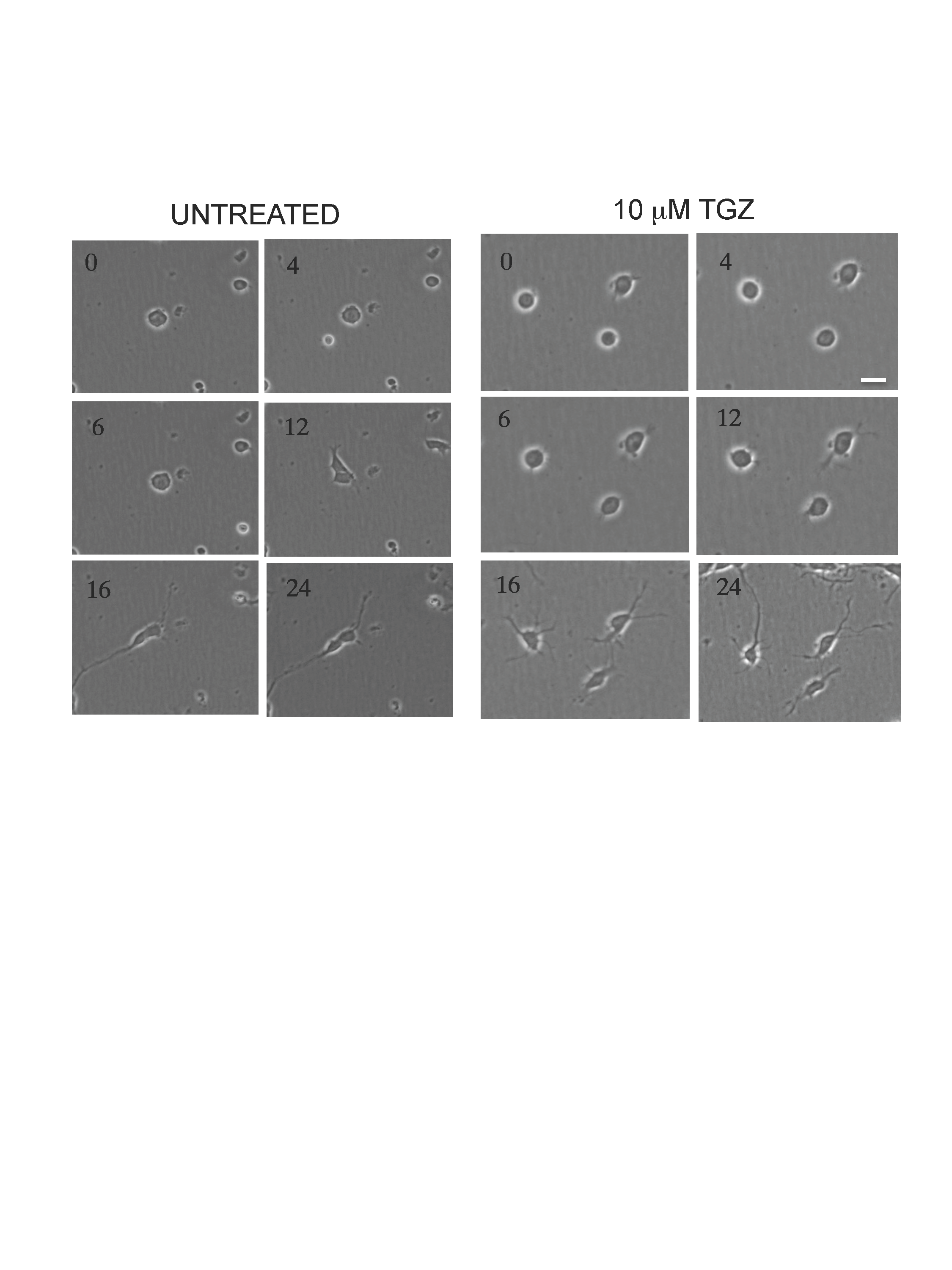

Supplement: Figure S1 — Troglitazone increases axonal elongation in hippocampal neurons. Hippocampal neurons recently plated were treated with 10 µM troglitazone (TGZ) and axonal development was observed by video microscopy. Neurons were mounted in a culture chamber controlling temperature, CO2, and humidity. Images were taken every hour using a cool CCD fluorescence camera (Zeiss, Germany). (TIFF) [file pone.0065140.s001.tiff]

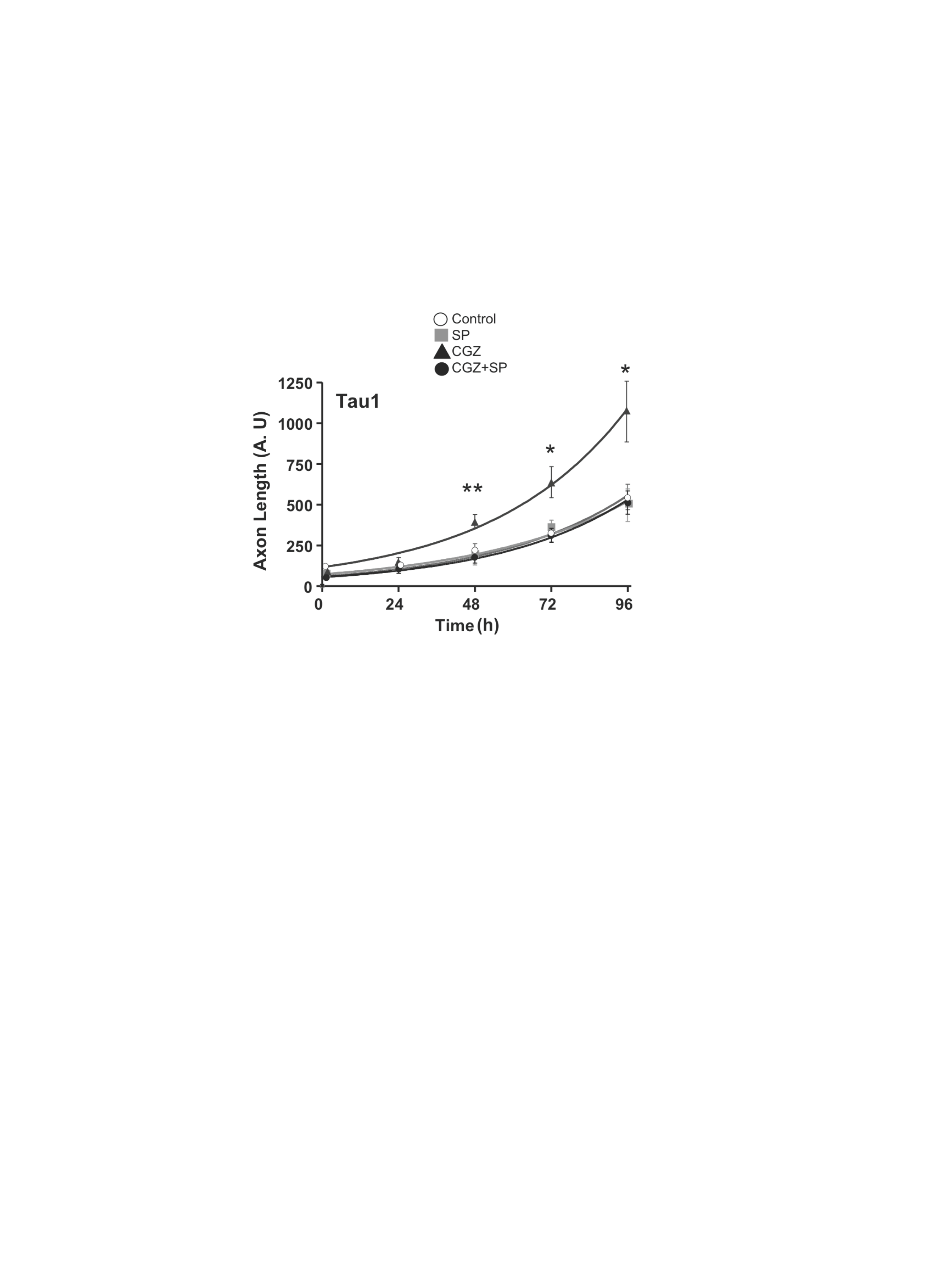

Supplement: Figure S2 — PPARγ activation increase of axonal elongation is mediated by JNK activation. Hippocampal neurons treated with CGZ, SP, and CGZ+SP were fixed at the indicated times and immunofluorescence against tau-1 was done. Axonal length was evaluated using Image Pro software. Data are the mean ± S.E.M. of 4 independent experiments, *p<0.05 and **p<0.01. (TIFF) [file pone.0065140.s002.tiff]
